# High-fat diet reveals the impact of *Sar1b* defects on lipid and lipoprotein profile and cholesterol metabolism

**DOI:** 10.1016/j.jlr.2023.100423

**Published:** 2023-08-07

**Authors:** Nickolas Auclair, Alain T. Sané, Léna Ahmarani, Nour-El-Houda Ould-Chikh, Nathalie Patey, Jean-François Beaulieu, Edgard Delvin, Schohraya Spahis, Emile Levy

**Affiliations:** 1 Research Center, CHU Sainte-Justine, Université de Montréal, Montreal, Quebec, Canada; 2 Department of Pharmacology & Physiology, Université de Montréal, Montreal, Quebec, Canada; 3 Laboratory of Intestinal Physiopathology, Department of Immunology and Cell Biology, Faculty of Medicine and Health Sciences, Université de Sherbrooke, Sherbrooke, Quebec, Canada; 4 Department of Nutrition, Université de Montréal, Montreal, Quebec, Canada

**Keywords:** *Sar1b* gene, chylomicron, intestinal fat malabsorption, lipoprotein composition, embryonic lethality, high-fat diet

## Abstract

Biallelic pathogenic variants of the *Sar1b* gene cause chylomicron retention disease (CRD) whose central phenotype is the inability to secrete chylomicrons. Patients with CRD experience numerous clinical symptoms such as gastrointestinal, hepatic, neuromuscular, ophthalmic, and cardiological abnormalities. Recently, the production of mice expressing either a targeted deletion or mutation of *Sar1b* recapitulated biochemical and gastrointestinal defects associated with CRD. The present study was conducted to better understand little-known aspects of *Sar1b* mutations, including mouse embryonic development, lipid profile, and lipoprotein composition in response to high-fat diet, gut and liver cholesterol metabolism, sex-specific effects, and genotype-phenotype differences. *Sar1b* deletion and mutation produce a lethal phenotype in homozygous mice, which display intestinal lipid accumulation without any gross morphological abnormalities. On high-fat diet, mutant mice exhibit more marked abnormalities in body composition, adipose tissue and liver weight, plasma cholesterol, non-HDL cholesterol and polyunsaturated fatty acids than those on the regular Chow diet. Divergences were also noted in lipoprotein lipid composition, lipid ratios (serving as indices of particle size) and lipoprotein-apolipoprotein distribution. *Sar1b* defects significantly reduce gut cholesterol accumulation while altering key players in cholesterol metabolism. Noteworthy, variations were observed between males and females, and between *Sar1b* deletion and mutation phenotypes. Overall, mutant animal findings reveal the importance of Sar1b in several biochemical, metabolic and developmental processes.

Lipid transport and metabolism are complex processes, but the development of cellular and molecular tools over the past two decades has greatly advanced our knowledge of the critical players and pathways involved. The two organs responsible for transporting hydrophobic lipids in the form of large macromolecules, known as lipoproteins, are the intestine and liver (1, 2, 3, 4). While dietary lipids are assembled with apolipoproteins (Apo)s in the enterocytes to form chylomicrons (CM), endogenous lipids are complexed with Apos to generate very low-density lipoproteins (VLDL). Both CM and VLDL are composed of a central lipid core consisting of triglycerides (TG) and cholesteryl ester (CE), which is surrounded by a free cholesterol (FC), phospholipid (PL) and Apo outer shell.

The biogenesis of CMs and VLDLs definitely requires the presence of critical proteins in the endoplasmic reticulum (ER) of both the enterocyte and hepatocyte. Investigation of genetic disorders, such as abetalipoproteinemia and hypobetalipoproteinemia, have been of immense support in defining the indispensable role of microsomal triglyceride transfer protein (MTTP) and Apo B-48 for the gut and Apo B-100 for the liver, respectively (5, 6, 7, 8, 9). A third congenital disorder, called chylomicron retention disease (CRD), has been identified subsequently, and is caused by mutations in the *Sar1b* gene, coding for a GTPase protein of the Ras superfamily. The Sar1b protein product is a part of the vesicular coat protein complex II, forming a shell around the vesicles that transport CM cargo in the secretory pathway (10, 11). It has been suggested that *Sar1b* mutations inhibit CM trafficking between the ER and Golgi, resulting in CM secretion, thus causing vitamin and essential fatty acid deficiency in patients.

Quite recently, we generated a mouse expressing either a targeted deletion or mutation similar to that in human *Sar1b* in CRD using the clustered regularly interspaced short palindromic repeats (CRISPR)-Cas9 system (12). Heterozygous mice exhibited the gastrointestinal abnormalities observed in CRD patients, including fat intestinal malabsorption, steatorrhea, CM secretion failure, hypocholesterolemia, and hypoalphalipoproteinemia (12). Moreover, these genetically modified *Sar1b* mice displayed disturbed intestinal lipid homeostasis as reflected by elevated fatty acid β-oxidation and diminished lipogenesis in association with powerful transcription factors (12).

Despite great achievements accomplished to date, additional efforts are still necessary to improve our understanding of CRD disorders. Among the aspects that need to be clarified, there are the following questions: *i*) What is the importance of Sar1b in mouse embryonic development?; *ii*) How *Sar1b* heterozygosity respond to a high-fat diet (HFD) in terms of lipid profile and lipoprotein composition?; *iii*) Do genetic *Sar1b* defects affect cholesterol (CHOL) metabolism in the gut and liver?; *iv*) Does disruption of Sar1b contribute to sex disparities?; and *v*) Are *Sar1b* mutation and deletion characterized by genotype-phenotype differences? The objective of the present work is precisely to focus on these different issues using mouse models.

## Materials and methods

### Mouse models

CRISPR-Cas9 technology was used to obtain heterozygous male and female *Sar1b* deletion (*Sar1b*^del/+^) and *Sar1b* mutation (*Sar1b*^mut/+^) mice on a C57BL/6N background. The detailed steps for the generation and confirmation of these two mouse models were described previously (12). Briefly, *Sar1b*^del/+^ mice were developed by generating a 545 bp deletion in exon 2 of *Sar1b* on chromosome 11 (GRCm38/mm10, chr11: 51, 777, 181-51, 777, 725 Del) whereas for *Sar1b*^mut/+^ mice, a G-to-A substitution was inserted in exon 2 on chromosome 11 (GRCm38/mm10, chr11: 51, 789, 257G>A) to replace aspartic acid with asparagine amino acid at the position 137 (p.D137N).

For embryonic experiments, heterozygous male and female mice (*Sar1b*^del/+^ and *Sar1b*^mut/+^) were mated. As soon as a vaginal plug was detected, the female was separated from the male and considered pregnant (day E0.5). At E9.5, E13.5, and E18.5, the female was euthanized by exposure to CO_2_, and the embryos were removed. The intestine, liver, and brain of the embryos at E18.5 were harvested for histology and/or flash frozen for future experiments. Similarly, the yolk sacs of E13.5 embryos were also harvested for histology.

### Animal experiments

Heterozygous mice were fed a chow diet (Teklad 18% protein diet, Harlan Laboratories, Envigo) or a HFD (60% fat Bio-serv F3282, Bio-serv) for 8 weeks. Mice were housed individually in a controlled environment (22°C and a 12-h daylight cycle) while food and water were given ad libitum. Body weight was evaluated twice a week. The mice were fasted for 6 h, anesthetized with isoflurane, and euthanized by heart puncture. Plasma was isolated with EDTA (1 mg/ml) from blood by 10 min centrifugation at 2,000 *g*, and different organs were removed, flash frozen in liquid nitrogen, and stored at −80°C. All procedures were approved by the Institutional Animal Care Committee of the Sainte-Justine Hospital Research Center.

### Biochemical analysis

Plasma TG, total CHOL, and FC were analyzed with commercial colorimetric kits (Wako Diagnostics). Insulin was determined with a commercial ELISA kit (Mercodia, Sweden). Blood glucose was measured with a glucometer (Contour Next, Bayer). The homeostatic model assessment for insulin resistance IR (HOMA-IR) was calculated with the formula: insulin (mIU/ml) × glucose (mM))/22.5. HDL-CHOL was isolated from plasma by precipitation with polyethylene glycol 6000. To this end, polyethylene glycol was mixed in a 1:1 ratio with 10 μl of plasma. This mixture was left to stand at room temperature for 20 min, then CHOL was measured in the top layer of the sample after centrifugation at 4,600 rpm for 40 min (12).

### Lipoproteins isolation

Isolation of VLDL, low density lipoprotein (LDL), and HDL was performed as previously described (13). Pooled plasma samples from 3 to 5 mice of the same group were subjected to a discontinuous sequential gradient centrifugation at 4°C in a Beckman LE-80 ultracentrifuge. To this end, KBr solution was layered on top of the plasma sample allowing the lipoproteins to float according to their specific density. First, CMs were floated at 25,000 *g* for 30 min with a SW 55 Ti rotor at a density of 1.006 g/ml (Beckman Coulter). Subsequently, VLDL and LDL were isolated at 40,000 *g* for 18 h with a Ti 70.1 rotor (Beckman Coulter) at gradients of 1.006 g/ml and 1.063 g/ml, respectively. Finally, HDLs were isolated at 1.21 g/ml with the same Ti 70.1 rotor at 40,000 *g* for 48 h. After isolation, lipoproteins were washed with a buffer of the same salt density and dialyzed using a buffer containing 0.15 M NaCl and 0.001 M EDTA at pH 7.

### Lipoprotein composition and apolipoprotein analysis

Protein and lipid compositions of each lipoprotein fraction were analyzed. TG, CHOL, and FC were measured with the kits specified above. The CE moiety was calculated by subtracting FC from total CHOL. PLs were measured with Bartlett's method (14) while proteins were quantified by the Bradford method using Bio-Rad Protein Assay Dye (Bio-Rad). The Bartlett’s technique was employed for the colorimetric determination of inorganic phosphate (14). Briefly, 70% perchloric acid was added to isolated fractions, which was heated at 150°C for 3 h. Then, the tubes were cooled to room temperature and topped up with water, ammonium molybdate, and aminonaphthalenesulfonic acid. After boiling, for 15 min at 100°C, the absorbance of the mixture was measured at 830 nm.

As for Apo evaluation, lipoproteins were loaded into a 4%–20% SDS-PAGE stain-free gel (Bio-Rad). Using the Bio-Rad's new Stain-Free technology (15), the densitometric distribution of Apos on the gels was estimated with the Image Lab software (https://www.bio-rad.com/fr-ca/product/image-lab-software?ID=KRE6P5E8Z) after image acquisition with the ChemiDoc MP (Bio-Rad). The proportion of specific Apos was defined as a percent of total Apo content.

### Intestinal and liver lipid analysis

Lipids from approximately 25–30 mg of the homogenized jejunum and liver in EDTA buffer were extracted overnight in a Folch solution (chloroform/methanol, 2:1) at 4°C. After centrifugation at 2,500 *g* for 15 min, the lower phase was dried under nitrogen at 50°C, and the lipids were resuspended in 400 μl of water. Total TG and CHOL were determined with the same commercial kits.

### Fatty acid composition

Plasma fatty acids (FA) were extracted by transesterification as described previously (16). Briefly, an internal standard (C19:1) dissolved in hexane:methanol (1:4) and an acetyl chloride solution were added to plasma (100 μl), which was heated (100°C) for 1 h. After cooling, 6% K_2_CO_3_ was added to the solution and centrifuged at 3,000 *g* for 10 min. FAs from the hexane upper phase were separated by gas chromatography equipped with a flame ionization detector (7890A, Agilent Technologies) (16). FAs of the different samples were identified and calculated based on the retention times of known FA standards. The area under the curve of the FAs was calculated with the Agilent ChemStation (Agilent Technologies).

### Tissue histology

The brain, liver, and intestine specimens were fixed in 10% formalin overnight and then embedded in paraffin. Sections of the liver and intestine (3 μm) and the brain (7 μm) were obtained with a microtome, stained with hematoxylin-eosin, and examined by light microscopy. Images of the stained tissues were captured with a Zeiss Imager A1 and measurements were carried out with the AxioVision software (https://www.micro-shop.zeiss.com/en/us/system/software+axiovision-axiovision+program-axiovision+software/10221/#variants). The yolk sac was fixed in 10% formalin and embedded with optimal cutting temperature compound. Tissues were cryosectioned (4 μm) with a cryostat and stained with hematoxylin and Oil Red O (Sigma-Aldrich). Total lipid surface area and number of particles, per microscopic field, were evaluated with Image J software (NIH) and calculated as previously described using invert lookup tables followed by the addition of a specific threshold (17). Villi were surrounded to avoid measuring the muscular section of the intestine.

### RNA isolation and reverse transcription-quantitative PCR analysis

The liver and jejunum were homogenized and RNA was extracted using Trizol according to the manufacturer's instructions (Ambion, Thermo Fisher Scientific). RNA concentration was measured using a BioDrop spectrophotometer (NanoDrop 8000, Thermo Fisher Scientific) while the absorbance ratio at 260 and 280 nm was used to determine purity (18, 19). The complementary DNA was generated by reverse transcription with the All-In-One 5X RT Master Mix from Applied Biological Materials and gene expression was amplified with PowerUP SYBR Green Master Mix (Life Technologies) using the 7500 Fast Real-Time PCR System (Applied Biosystems). The thermal profile for quantitative PCR was as follows: initial denaturation at 95°C for 10 min, 40 cycles of denaturation at 95°C for 15 s, and annealing and extension at 60°C for 1 min. Relative mRNA expression for each gene was obtained by the 2^−ΔΔCT^ calculation method (20). Primers used for the experiments are listed in the supplementary materials (supplemental Table S1).

### Statistical analysis

For the embryonic experiments, data were analyzed by a one-way ANOVA and differences between means were determined by the Tukey post hoc test. For adult mice, a two-way ANOVA was used for experiments with diet and genetic background as two independent variables. Dunnett's post hoc multiple comparisons test was chosen to compare test groups with their respective controls. GraphPad Prism 8.0 software (GraphPad, https://www.graphpad.com/features) was used for all statistical analyses. Results are presented as means ± SEM and significance is considered at *P* < 0.05.

## Results

### Embryonic development and tissue histology of *Sar1b*-altered embryos

Murine models for CRD were successfully developed. Both heterozygous *Sar1b*^del/+^ and *Sar1b*^mut/+^ mice were phenotypically healthy and fertile. To confirm and extend the data of our recent study (12), these heterozygous mice were intercrossed for several generations. Weaned offspring genotyping revealed no homozygous *Sar1b*^del/del^ and *Sar1b*^mut/mut^ mice, suggesting that *Sar1b* defects could cause embryonic lethality (Table 1). To delineate if homozygous *Sar1b* induces embryonic lethality, we examined embryos at embryonic days 9.5, 13.5, and 18.5. In contrast to our hypothesis, the presence of homozygous embryos (*Sar1b*^del/del^ and *Sar1b*^mut/mut^) was noticed at the three stages of development.

**Table 1:** Genotypes of offspring from intercrosses of Sar1b^del/+^ mice and Sar1b^mut/+^ mice

Sar1b genotype						
Genotypes of Embryos or Postnatal mice from Intercross of heterozygous *Sar1b*^del/+^ mice						
Mating	Litters	Average pups/litter	Total pups	+/+	del/+	del/del
Postnatal	21	6.28	132	39 (29.5%)	86 (65.2%)	7^a^ (5.3%)
E18.5	4	7.75	31	8 (25.8%)	13 (41.9%)	10 (32.3%)
E13.5	3	7.33	22	8 (36.36%)	7 (31.81%)	7 (31.81%)
E9.5	2	10	20	0 (0%)	19 (95%)	1 (5%)

Genotypes of embryos or postnatal mice from intercross of heterozygous *Sar1b*^mut/+^ mice						
Mating	Litters	Average pups/litter	Total pups	+/+	mut/+	mut/mut
Postnatal	35	6.11	214	129 (60.3%)	85 (39.7%)	0
E18.5	3	9.33	28	9 (32.1%)	15 (53.6%)	4 (14.3%)
E13.5	3	7.66	23	5 (21.7%)	14 (60.9%)	4 (17.4%)
E9.5	3	8	24	8 (33.3%)	12 (50%)	4 (16.7%)

Male and female heterozygous *Sar1b*^del/+^ and *Sar1b*^mut/+^ mice were mated. Part of the tail of postnatal mice was collected and genotyped. Regarding embryos, as soon as a vaginal plug was found on female mice, they were considered pregnant at day E0.5. After 9, 13, or 18 days, female mice were euthanized with a CO_2_ stream and the embryos were collected. A portion of the yolk sac was removed for genotyping. The number of litters, average pups per litter, total number of pups and number of pups for each genotype were calculated.

a Pups were dead at birth.

**Fig. 1:**
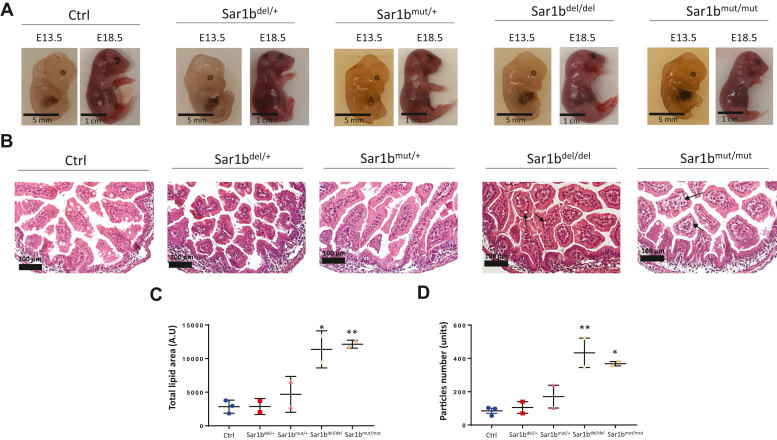
Embryonic development and tissue histology of *Sar1b*-altered embryos. Heterozygous male and female *Sar1b*^del/+^ and *Sar1b*^mut/+^ mice were mated. As soon as a vaginal plug was found on the female mice, they were considered pregnant at day E0.5. After 13 or 18 days, the female mice were euthanized with a CO_2_ stream and the embryos were collected. Photographs of the embryos at these two different time points were taken (A) and the intestines were isolated from the embryos at E18.5, fixed with formalin, embedded in paraffin, and stained with hematoxylin and eosin. Photographs of these intestines were taken under the microscope (B) and the vesicles are indicated by black arrows. The total lipid surface area (C), as well as the number of lipid particles (D), were analyzed with image j software by applying a specific threshold, followed by the use of the particle analysis tool. Results represent means ± SEM of at least two embryos. ^∗^*P* < 0.05, ^∗∗^*P* < 0.01, versus controls (ctrl).

The morphological examination of these homozygous embryos did not disclose any macroscopic abnormalities compared to control embryos (Fig. 1A). The *Sar1b* mutated or deleted embryos did not appear growth retarded or less developed. Attention was then shifted to the organs of these embryonic homozygotes. The morphology of the brain, liver, and intestine of 18.5-days embryos revealed to be normal (supplemental Fig. S1). We then turned to the yolk sac membrane of 13.5-days embryos and no apparent lipid alterations were observed following Oil Red O staining (supplemental Fig. S1), and they showed similar localization and labeling of MTTP and ApoB (supplemental Fig. S2). Nevertheless, histology of the intestine showed a marked increase in white vesicles in homozygous mice that were not present in controls, which could be attributed to lipid accumulation (Fig. 1B). Analysis of the histological photographs with Image J software confirmed a significant increase in total lipids surface area (Fig. 1C) and particles number (Fig. 1D) in both homozygous mouse lines compared to controls.

### Anthropometric parameters and biochemical analysis of plasma from *Sar1b*^del/+^ and *Sar1b*^mut/+^ mice

**Fig. 2:**
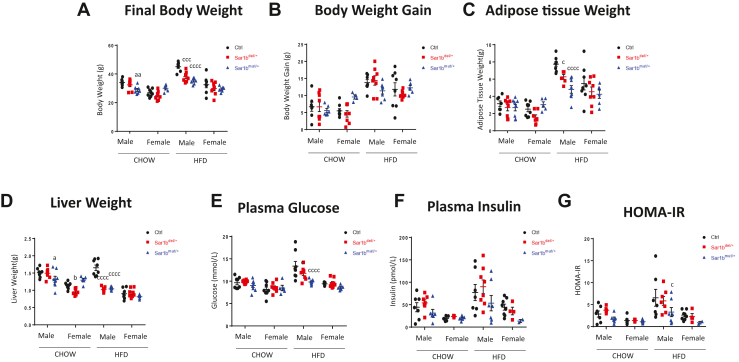
Impact of *Sar1b* mutation or deletion on anthropometric and insulin resistance parameters. Five-week-old male and female controls (ctrl), *Sar1b*^del/+^, and *Sar1b*^mut/+^ mice were fed a Chow or a high-fat diet (HFD) for 8 weeks. After a 6-h fast, the body weight (A) of the mice was taken and the body weight gain was calculated (B) just before the mice were euthanized by cardiac puncture. The weight of adipose tissue (C) and liver (D) was recorded, the tissues were flash frozen, and plasma was isolated from blood. Plasma glucose (E) was analyzed with a glucometer while insulin (F) was analyzed in plasma with commercial kits. HOMA-IR (G) was calculated with the previous plasma parameters. Results represent means ± SEM of at least five mice. a = *P* < 0.05, aa = *P* < 0.01, aaa = *P* < 0.001 versus Chow ctrl male; b = *P* < 0.05 versus CHOW ctrl female; c = *P* < 0.05, ccc = *P* < 0.001; cccc = *P* < 0.0001 versus HFD ctrl male. HOMA-IR, homeostatic model assessment-insulin resistance.

Since homozygous mice do not develop after birth and that heterozygous mice on the other hand do thrive, we decided to study how heterozygous mice with *Sar1b* aberrations cope with a HFD. Animals were given either a 60% fat or a regular Chow diet for 8 weeks. Overall, control mice fed a HFD had a higher final body weight (Fig. 2A, B), displayed more adipose tissue (Fig. 2C), had a larger liver (Fig. 2D), and developed IR as reflected by the plasma insulin and homeostatic model assessment-IR (Fig. 2E–G). These differences in anthropometric and IR parameters between Chow and HFD were much more pronounced in males than in females. On their part, heterozygotes, especially those with the *Sar1b* mutation subjected to the control Chow diet, showed a slight downward trend of anthropometric parameters and IR compared to controls. However, changes in anthropometric and IR parameters were more important in heterozygotes on HFD compared to controls, which indicates a significant interaction. Importantly, the results were more consistent for the *Sar1b*^mut/+^ than for the *Sar1b*^del/+^ mice and were significantly different only for males and not for females.

**Fig. 3:**
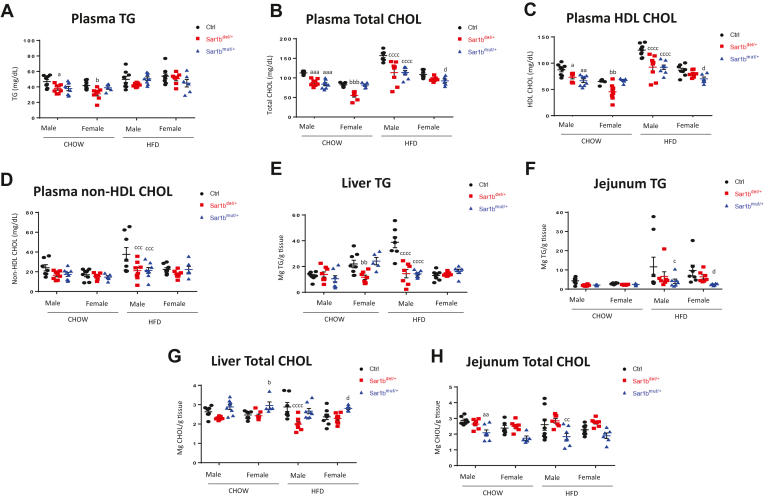
Effects of *Sar1b* mutation or deletion on plasma and tissues lipid parameters. Five-week-old male and female controls (Ctrl), *Sar1b*^del/+^, and *Sar1b*^mut/+^ mice were fed a Chow or a high-fat diet (HFD) for 8 weeks. After a 6-h fast, mice were euthanized by cardiac puncture, tissues were flash frozen, and plasma was isolated from blood. Plasma triglycerides (TG) (A) and total cholesterol (CHOL) (B) were analyzed in plasma with commercial kits. As for HDL, it was isolated by precipitation with polyethylene glycol 6000 (C), while non-HDL-CHOL (D) was calculated with the previous plasma parameters. As for lipids in the tissues, flash-frozen livers and jejunum of male and female mice were extracted with Folch (2:1 chloroform/methanol). After drying the lipids, they were resuspended in H_2_O for TG (E, F) and CHOL (G, H) analysis by commercial kits. Results represent means ± SEM of at least five mice. a = *P* < 0.05, aa = *P* < 0.01, aaa = *P* < 0.001 versus Chow ctrl male; b = *P* < 0.05, bb = *P* < 0.01, bbb = *P* < 0.001 versus Chow ctrl female; c = *P* < 0.05, cc = *P* < 0.01, ccc = *P* < 0.001, cccc = *P* < 0.0001 versus HFD ctrl male; d = *P* < 0.05, dddd = *P* < 0.0001 versus HFD ctrl female.

With regards to lipid parameters on the control Chow diet, the genetically modified mice had lower plasma TG and CHOL concentrations compared to controls (Fig. 3A, B). Although levels of these lipids increased in heterozygotes on the HFD compared with the Chow diet, the elevation was more restricted than in controls. Similar results characterize HDL-CHOL and non-HDL-CHOL (Fig. 3C, D). Again, differences were observed between the *Sar1b*^mut/+^ and *Sar1b*^del/+^ mice and between male and female mice. Likewise, when lipids were examined in the liver and intestine, genetic *Sar1b* aberrations limited the magnitude of elevations when switching from the regular diet to the HFD (Fig. 3E–H). Male–female differences were evidenced with respect to lipid accumulation in the jejunum and liver, particularly in the liver as females were unresponsive to the HFD.

**Fig. 4:**
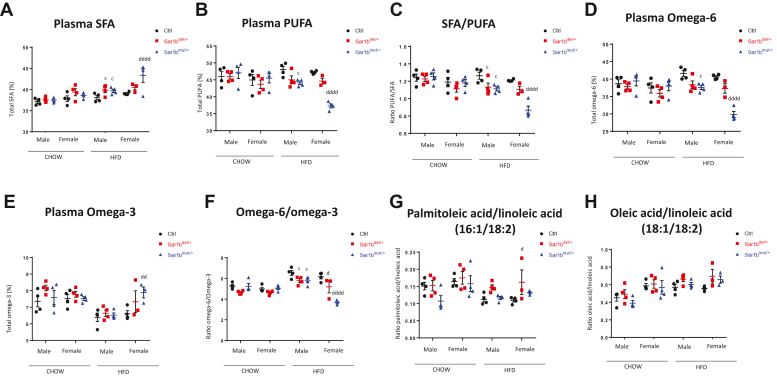
Effects of *Sar1b* alteration on plasma fatty acid composition. Five-week-old male and female controls (Ctrl), *Sar1b*^del/+^, and *Sar1b*^mut/+^ mice were fed a Chow or a high-fat diet (HFD) for 8 weeks. After a 6-h fast, mice were euthanized by cardiac puncture, tissues were flash frozen, and plasma was isolated from blood. Fatty acids were extracted from plasma by transesterification and separated by gas chromatography as detailed in the Materials and Methods section. Total saturated fatty acids (SFA) (A), polyunsaturated fatty acids (PUFA) (B), SFA/PUFA ratio (C), omega-6 (D) and omega-3 (E) are shown. Specific ratios of omega 6/omega-3 (F), palmitoleic acid/linoleic acid (16:1/18:2) (G) and oleic acid/linoleic acid (18:1/18:2) (H) are also demonstrated. Results represent means ± SEM of at least four mice; c = *P* < 0.05 versus HFD ctrl male; dd = *P* < 0.01, dddd = *P* < 0.0001 versus HFD ctrl female.

As the composition and incorporation of FA in plasma results from distinct processes, including intake, metabolism, and peripheral utilization, examination of the FA response to *Sar1b* genetic defects following a HFD challenge was necessary. Both controls and heterozygotes and males and females show similar FA composition on the regular Chow diet (Fig. 4A–H). However, divergences were obvious between controls and heterozygous mice on the HFD: male and female heterozygous mice had more saturated FA (SFA) (Fig. 4A), less polyunsaturated FA (PUFA) (Fig. 4B), a lower SFA/PUFA ratio (Fig. 4C), less omega-6 (Fig. 4D), no changes in omega-3 (Fig. 4E), but away smaller ratio of omega-6/omega-3 (Fig. 4F). A small increase was noted in the ratio of palmitoleic acid/linoleic acid (16:1/18:2) (Fig. 4G) while no changes were noted for the ratio oleic acid/linoleic acid (18:1/18:2) (Fig. 4H). These results were more prominent in *Sar1b*^mut/+^ than in *Sar1b*^del/+^ mice.

### Lipid and apolipoprotein composition of lipoproteins isolated from heterozygous Sar1b mice

The noticeable decrease in plasma CHOL in heterozygous mice and the fact that Sar1b protein is involved in lipoprotein secretion prompted us to analyze the composition of important plasma lipoproteins such as VLDL, LDL, and HDL. As shown in Table 2, the chemical composition of VLDL was altered in heterozygous mice. In particular, compared to controls, heterozygous mice under control Chow or HFD diet had less TG in VLDL, with the exception of *Sar1b*^mut/+^ which exhibited more VLDL-TG on HFD diet. As a consequence, changes were noted in VLDL weight ratio calculated as the ratio of core constituents (TG and CE) to surface constituents (FC + protein + PL). Regarding the composition of LDL and HDL, both heterozygous mice subjected to Chow or HFD diet showed an increase in their proteins, resulting in the reduction of the CE/protein ratio. However, this phenomenon was more predominant and significant in *Sar1b*^mut/+^ mice on the HFD diet. These results most likely indicate that the reduction in plasma CHOL concentration reflects a reduction in lipoprotein particles and not by lipoprotein size.

**Table 2:** Chemical composition of lipoproteins isolated from plasma of Sar1b^del/+^ and Sar1b^mut/+^ mice under Chow and high-fat diets

Lipoproteins	Diet	Genotype	Composition (%)					Weight Ratios			
			TG	CE	FC	PL	PR	TG/CE	CE/PL	CE/PR	(TG + CE)/(FC + PL + PR)
VLDL	CHOW	Ctrl	52.85 ± 4.32	2.68 ± 0.27	8.13 ± 0.30	18.24 ± 1.99	18.10 ± 2.52	20.0141 ± 3.1778	0.1483 ± 0.0204	0.1506 ± 0.0247	1.2686 ± 0.2079
		Sar1b^del/+^	30.32 ± 2.41∗∗∗∗	2.08 ± 0.42	5.50 ± 0.67∗	41.43 ± 0.38∗∗∗	20.68 ± 1.70	15.4371 ± 4.2427	0.0502 ± 0.0105	0.0995 ± 0.0119	0.4804 ± 0.0436∗∗∗
		Sar1b^mut/+^	45.36 ± 2.01	2.66 ± 0.26	8.00 ± 0.92	23.65 ± 4.64	20.34 ± 2.41	17.2875 ± 2.3564	0.1158 ± 0.0187	0.1330 ± 0.0220	0.9259 ± 0.0650∗
	HFD	Ctrl	47.34 ± 4.94	2.59 ± 1.04	4.87 ± 0.55	24.12 ± 3.29	21.07 ± 2.94	23.2828 ± 12.8931	0.1056 ± 0.0377	0.1277 ± 0.0524	1.0158 ± 0.1988
		Sar1b^del/+^	35.71 ± 2.58^$$^	4.18 ± 0.14^$^	3.93 ± 1.47	23.94 ± 6.73	32.23 ± 3.28^$$$^	8.5547 ± 0.7934	0.1908 ± 0.0598	0.1309 ± 0.0118	0.6667 ± 0.0678^$^
		Sar1b^mut/+^	56.54 ± 0.30^$^	3.01 ± 0.61	8.50 ± 0.18^$$$^	12.16 ± 2.26^$$^	19.8 ± 1.91	19.5487 ± 3.7149	0.2621 ± 0.0950^$$^	0.1517 ± 0.0243	1.4723 ± 0.0264^$$^
LDL	CHOW	Ctrl	10.96 ± 2.28	23.32 ± 1.63	11.06 ± 0.44	32.92 ± 3.41	21.74 ± 2.57	0.4767 ± 0.1273	0.7145 ± 0.0806	1.0925 ± 0.1822	0.5229 ± 0.0437
		Sar1b^del/+^	12.85 ± 2.54	20.62 ± 1.05	11.05 ± 0.40	27.24 ± 0.92	28.24 ± 1.21∗∗	0.6312 ± 0.1561	0.7564 ± 0.0143	0.7314 ± 0.0497∗∗	0.5040 ± 0.0361
		Sar1b^mut/+^	13.08 ± 2.28	20.02 ± 1.80	11.12 ± 0.85	31.36 ± 3.40	24.42 ± 2.73	0.6602 ± 0.1277	0.6464 ± 0.0949	0.8383 ± 0.1666	0.4974 ± 0.0621
	HFD	Ctrl	7.31 ± 1.29	23.32 ± 1.63	12.57 ± 0.63	37.69 ± 1.56	19.11 ± 1.12	0.3142 ± 0.0527	0.6212 ± 0.0644	1.2279 ± 0.1405	0.4432 ± 0.0476
		Sar1b^del/+^	7.18 ± 1.96	22.16 ± 1.21	11.44 ± 0.57	37.36 ± 3.11	21.85 ± 0.38	0.3245 ± 0.0855	0.5996 ± 0.0794	1.0137 ± 0.0389	0.4169 ± 0.0492
		Sar1b^mut/+^	9.01 ± 2.03	25.41 ± 0.75	12.99 ± 0.33	23.79 ± 2.85^$$$$^	28.79 ± 1.74^$$$$^	0.3569 ± 0.0899	1.0820 ± 0.1198^$$$$^	0.8850 ± 0.0480^$^	0.5257 ± 0.0350
HDL	CHOW	Ctrl	0.75 ± 0.08	22.14 ± 0.98	4.60 ± 0.29	32.52 ± 1.69	40.00 ± 1.31	0.0336 ± 0.0029	0.6834 ± 0.0553	0.5541 ± 0.0308	0.2970 ± 0.0171
		Sar1b^del/+^	0.66 ± 0.11	20.45 ± 1.04	4.42 ± 0.38	30.61 ± 3.06	43.86 ± 4.24	0.0320 ± 0.0036	0.6730 ± 0.0591	0.4724 ± 0.0683	0.2678 ± 0.0187
		Sar1b^mut/+^	0.73 ± 0.36	19.74 ± 1.84	4.80 ± 0.15	30.34 ± 0.91	44.39 ± 2.30	0.0388 ± 0.0217	0.6504 ± 0.0545	0.4478 ± 0.0653	0.2579 ± 0.0252
	HFD	Ctrl	1.14 ± 0.81	18.99 ± 1.01	4.91 ± 0.40	37.10 ± 2.17	37.87 ± 2.02	0.0579 ± 0.0386	0.5144 ± 0.0507	0.5038 ± 0.0461	0.2526 ± 0.0286
		Sar1b^del/+^	0.76 ± 0.41	19.14 ± 1.49	4.60 ± 0.36	35.34 ± 2.78	40.16 ± 2.29	0.0400 ± 0.0229	0.5454 ± 0.0626	0.4788 ± 0.0521	0.2488 ± 0.0231
		Sar1b^mut/+^	0.38 ± 0.05	17.29 ± 0.48	4.36 ± 0.22	33.19 ± 0.88	44.78 ± 0.84^$$^	0.0222 ± 0.0025	0.5214 ± 0.0226	0.3863 ± 0.0134^$^	0.2147 ± 0.0076

Plasma lipoproteins were separated by discontinuous density gradient ultracentrifugation. After preliminary centrifugation to remove chylomicrons (CM), very low density (VLDL), low density (LDL), and high density (HDL) lipoproteins were isolated at densities of 1.006 g/ml, 1.063 g/ml, and 1.210 g/ml, respectively.

Results represent the means ± SEM of 3–4 pooled plasma from mice. Data were analyzed using two-way ANOVA with the Dunnett post hoc test. ^∗^*P*< 0.05, ^∗∗^*P*< 0.01, ^∗∗∗^*P*< 0.001 versus CHOW Ctrl; ^$^*P*< 0.05, ^$$^*P*< 0.01, ^$$$^*P*< 0.0001, ^$$$$^*P*< 0.0001 versus HFD Ctrl.

CE, cholesteryl ester; FC, free cholesterol; HFD, high-fat diet; PL, phospholipids; PR, proteins; TG, triglycerides.

**Fig. 5:**
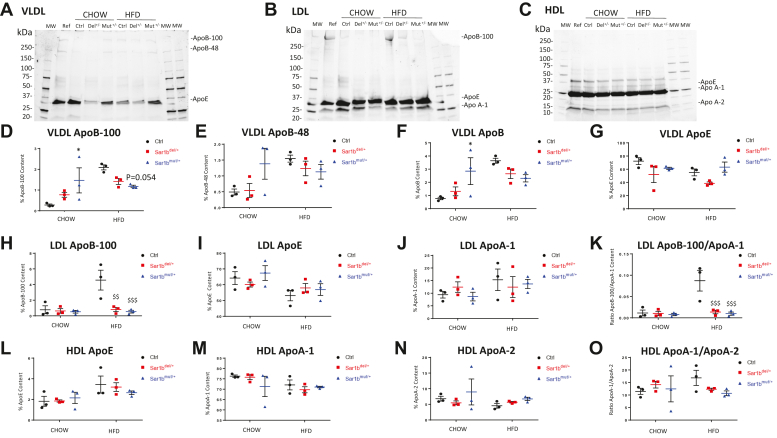
VLDL and LDL fractions of *Sar1b*^del/+^ and *Sar1b*^mut/+^ on HFD diet have less ApoB-100 than controls. Five-week-old male and female controls (Ctrl), *Sar1b*^del/+,^ and *Sar1b*^mut/+^ mice were fed a Chow or a high-fat diet (HFD) for 8 weeks. After a 6-h fast, mice were euthanized by cardiac puncture and plasma was isolated from blood. Plasma from 3 to 5 mice was pooled and subjected to sequential gradient centrifugation to isolate VLDL, LDL, and HDL. Lipoprotein fractions were loaded into a 4%–20% SDS-PAGE gel with Bio-rad stain-free technology. Apolipoprotein (Apo) bands were directly analyzed with Bio-rad Chemidoc and the percentage of a specific Apo was calculated based on the total Apo content. Example gels are given for VLDL (A), LDL (B), HDL (C), and results for Apos are given for Apo B-100 (D), Apo B-48 (E), total Apo B (F), Apo E (G) for VLDL, Apo B-100 (H), Apo E (I), Apo A-1 (J) Apo B-100/Apo A-1 ratio (K) for LDL, ApoE (L), ApoA-1 (M), ApoA-2 (N) and ApoA-1/ApoA-2 ratio (O) for HDL. Results represent means ± SEM from three pools of mouse plasma. Data were analyzed using 2-way ANOVA with Dunnett's post hoc test. ^∗^*P* < 0.05 versus Chow Ctrl; ^$$^*P* < 0.01, ^$$$^*P* < 0.001 versus HFD Ctrl.

**Fig. 6:**
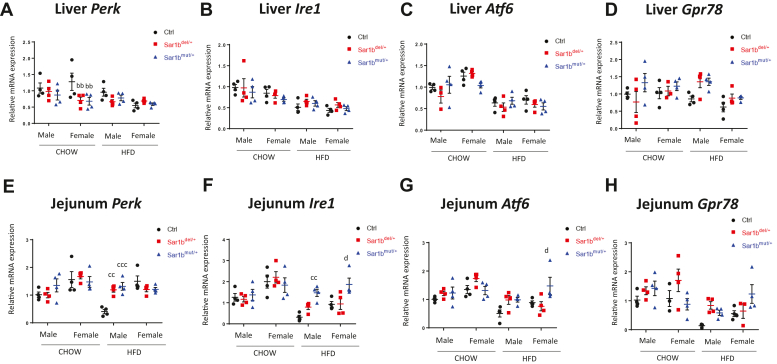
*Sar1b* defect affects reticulum endoplasmic stress. Five-week-old male and female controls (Ctrl), *Sar1b*^del/+^, and *Sar1b*^mut/+^ mice were fed a Chow or a high-fat diet (HFD) for 8 weeks. After a 6-h fast, mice were euthanized by cardiac puncture, and tissues were collected and flash-frozen. Liver and intestinal mRNAs were extracted with Trizol and cDNAs were generated by reverse transcription. For both tissues, PERK, IRE1, GPR78, and ATF6 were amplified by qRT-PCR (A–D) for the liver and (E–H) for the intestine. Results represent means ± SEM from at least four mice. bb = *P* < 0.01 versus female Chow ctrl. cc = *P* < 0.01, ccc = *P* < 0.001 versus male HFD ctrl; d = *P* < 0.05 versus female HFD ctrl. ATF6, activating transcription factor 6; cDNA, complementary DNA; GPR78, G protein-coupled receptor 78; IRE1, inositol-requiring enzyme 1; PERK, protein kinase RNA-like ER kinase.

Apo content was also quantified using SDS-PAGE for these three types of lipoproteins, and representative gel images are shown in Figure 5, Figure 6A–C. VLDL and LDL were characterized by a drop in ApoB-100 content in *Sar1b*^mut/+^ and *Sar1b*^del/+^ on HFD compared with their respective controls (Fig. 5D, H). This reduction was drastic for LDL (Fig. 5H) and caused a large drop in the Apo B-100/Apo A-1 ratio (Fig. 5K). As for the other Apos, no significant differences were noted in Apo B-48 and ApoE content of VLDL (Fig. 5E, F), Apo E and Apo A-1 content of LDL (Fig. 5I, J), and ApoE, Apo A-1 and Apo A-2 content of HDL (Fig. 5L–O) between controls and heterozygous mice on Chow or HFD.

### *Sar1b* defects and ER stress

**Fig. 7:**
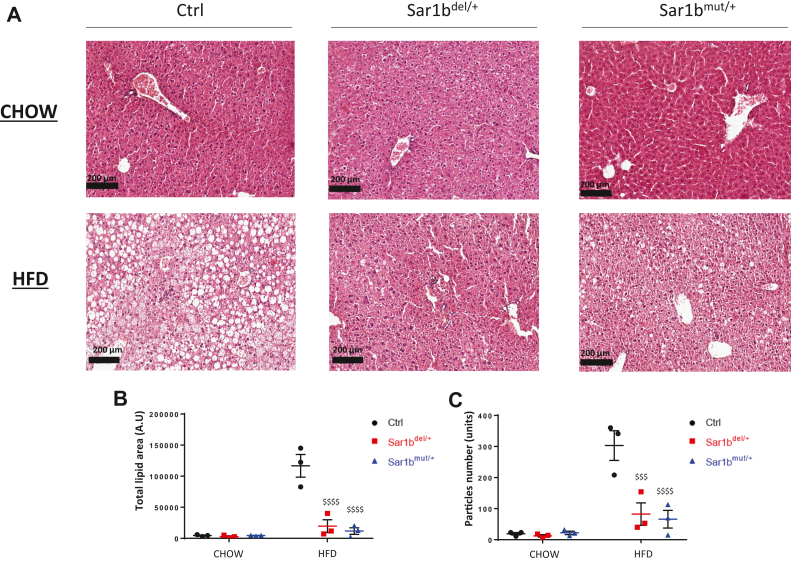
Histological lipids accumulation in the liver of *Sar1b*-altered mice. Five-week-old male controls (Ctrl), *Sar1b*^del/+,^ and *Sar1b*^mut/+^ mice were fed a Chow or a high-fat diet (HFD) for 8 weeks. After a 6-h fast, mice were euthanized by cardiac puncture, and tissues were fixed with formalin or flash frozen. For histology, after fixation, liver tissues from male mice were embedded in paraffin, stained with hematoxylin and eosin, and photographs were taken (A). Total lipid surface area (B) as well as the number of lipid particles (C) were analyzed with image j software by applying a specific threshold, followed by the use of the particle analysis tool. Results represent means ± SEM from three mice. ^$$$^*P* < 0.001, ^$$$$^*P* < 0.0001 versus HFD Ctrl.

Experiments were performed to assess whether defects in *Sar1b* promote ER stress. Our findings show a decreased gene expression of protein kinase RNA-like ER kinase *(Perk)* in the liver of *Sar1b*^mut/+^ and *Sar1b*^del/+^ females on the control Chow diet (Fig. 6A). On the other hand, no change was observed for activating transcription factor 6 (*Atf6*) and inositol-requiring enzyme 1 (*Ire1*) (Fig. 6B, C), while the expression of G protein-coupled receptor 78 (*Gpr78*) slightly raised for both sexes and genotypes in mice on a HFD diet (Fig. 6D). The results in the gut documented increased gene expression of *Perk*, *Ire1*, *Atf6* and *Gpr78* for mutant mice on the HFD compared to their respective controls (Fig. 7E–H).

### *Sar1b* defects and liver TG accumulation

Because HFD increases the development of hepatic steatosis, it was important to evaluate TG accumulation in the liver of *Sar1b*^mut/+^ and *Sar1b*^del/+^ mice in response to HFD. As was the case for liver weight, we have previously observed that hepatic TG of male heterozygous *Sar1b* mice on HFD was significantly lower than that of their respective controls (Fig. 3E). Confirmation was obtained with histology (Fig. 7A). Analysis of histological photographs actually revealed a lower abundance of total lipid surface area (Fig. 7B) and lipid particle number (Fig. 7C).

### *Sar1b* defects and cholesterol metabolism in the liver and intestine

**Fig. 8:**
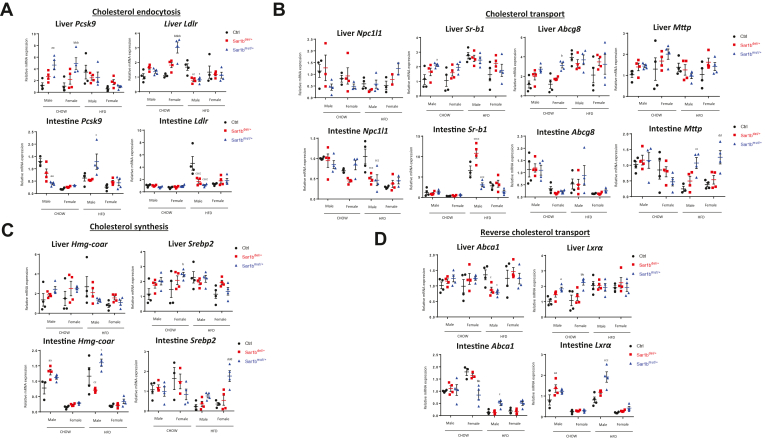
Alterations in *Sar1b* alter the expression of key genes involved in the liver and gut cholesterol metabolism. Five-week-old male and female controls (Ctrl), *Sar1b*^del/+^, and *Sar1b*^mut/+^ mice were fed either a Chow or a high-fat diet (HFD) for 8 weeks. After a 6-h fast, mice were euthanized by cardiac puncture, and tissues were collected and flash-frozen. The liver and intestinal mRNAs were extracted with Trizol and cDNAs were generated by reverse transcription. For both tissues, the genes for cholesterol endocytosis (A), cholesterol transport (B), cholesterol synthesis (C), and reverse cholesterol transport (D) were amplified by qPCR. Results represent means ± SEM from four mice. a = *P* < 0.05, aa = *P* < 0.01 versus Chow ctrl male. b = *P* < 0.05, bb = *P* < 0.01, bbb = *P* < 0.001, bbbb = *P* < 0.0001 versus Chow female ctrl; c = *P* < 0.05, cc = *P* < 0.01, ccc = *P* < 0.001, cccc = *P* < 0.0001 versus HFD male ctrl; dd = *P* < 0.01, dddd = *P* < 0.0001 versus HFD female ctrl. cDNA, complementary DNA; qPCR, quantitative PCR.

The alterations of CHOL concentrations in plasma and lipoproteins could imply a modification of intestinal and/or hepatic CHOL metabolism in heterozygous *Sar1b* mice. We therefore analyzed CHOL accumulation in these organs along with the gene expression of important CHOL metabolism biomarkers. As we have seen previously, slight increase in liver CHOL content was apparent in female *Sar1b*^mut/+^ mice subjected to HFD, as opposed to the reduction in male *Sar1b*^del/+^ mice compared with their respective controls (Fig. 3G). On the other hand, a consistent reduction in CHOL content was observed in the gut of male and female *Sar1b*^mut/+^ mice subjected to both diets, though more pronounced in male mice under a HFD (Fig. 3H). PCR analysis was performed to assess the expression of several important genes involved in CHOL endocytosis [proprotein convertase subtilisin/kexin type 9 serine protease (*Pcsk9*)], low density lipoprotein receptor (*Ldlr*) (Fig. 8A), transport [Niemann–Pick C1-like 1 (*Npc1l1*), scavenger receptor class B type I (*Sr-b1*), ATP-binding cassette transporters G8 (*Abcg8*), *Mttp*] (Fig. 8B), synthesis [3-hydroxy-3-methylglutaryl coenzyme A reductase (*Hmg-Coar*), sterol regulatory element-binding protein-2 (*Srebp2*)] (Fig. 8C) and reverse CHOL transport [ATP-binding cassette A1 (*Abca1*), Liver X receptor α (*Lxrα*)] (Fig. 8D). Notable differences were noted between control and heterozygous mice, liver and intestine, *Sar1b*^del/+^ and *Sar1b*^mut/+^ genotypes, males and females under the two diets.

## Discussion

Animal models have provided undeniable contributions to basic medical understanding and advancement. In the present study, we have developed mice with targeted defects similar to human *Sar1b* aberrations to gain more insight into CRD pathophysiology. Our findings (summarized in Table 3) pointed out the repercussions of *Sar1b* mutation or deletion, including embryonic alterations, lipid profiles and lipoprotein composition abnormalities, cholesterol metabolism inconsistencies, IR and ER stress.

**Table 3:** Summary of the impact of each mutation on the different general result areas

Characteristics	CHOW diet		High-fat diet	
	Sar1b^del/+^	Sar1b^mut/+^	Sar1b^del/+^	Sar1b^mut/+^
Anthropometric and biochemical parameters				
Weight	ND	ND	↓	↓
Insulin resistance	ND	ND	ND	↓
Liver steatosis	ND	ND	↓	↓
Intestinal steatosis	ND	ND	ND	↓
Hyperlipidemia	↓	↓	↓	↓
Essential fatty acids deficiency	ND	ND	↑	↑
Lipoprotein composition				
Lipoprotein cholesterol ester/proteins weight ratio	ND	ND	ND	↓
Apo B content	ND	ND	↓	↓
Endoplasmic reticulum stress markers				
Intestinal endoplasmic stress	ND	ND	↑	↑
Liver endoplasmic stress	ND	ND	ND	ND
Cholesterol metabolism markers				
Intestinal cholesterol endocytosis	ND	ND	↓	↓
Liver cholesterol endocytosis	ND	↑	ND	ND
Intestinal cholesterol transport	ND	ND	↓↑	↓↑
Liver cholesterol transport	ND	ND	ND	ND
Intestinal cholesterol synthesis	ND	ND	ND	↑
Liver cholesterol synthesis	ND	ND	ND	ND
Intestinal reverse cholesterol transport	ND	ND	ND	↑
Liver reverse cholesterol transport	ND	ND	ND	ND

ND, No difference; ↓, decrease; ↑, increase; ↓↑, increase or decrease in different genes.

Given our poor understanding of the interplay between *Sar1b* aberrations and fat feeding-mediated metabolism, we challenged *Sar1b*^mut/+^ and *Sar1b*^del/+^ mice with long-term HFD. The outcomes of these experiments would be all the more important in order to better appraise the recommendations to CRD patients to abstain from fat consumption. In addition, these experimental steps will help test the hypothesis that mice with *Sar1b* defects are protected from HFD-induced weight gain and metabolic abnormalities. As validated by our results, this assumption is largely due to the fact that the mutant mice showed some protection against weight gain, adipose tissue expansion, IR, hyperlipidemia and hepatic steatosis in a gender and genotype-dependent manner. Our findings may indicate a novel link between Sar1b-mediated lipid absorption and metabolism, which is supported by our previous work pointing out that transgenic mice overexpressing *Sar1b* developed a rise in body weight, adiposity, hepatic steatosis, plasma lipids and insulin insensitivity (21).

Our recent study has reported that mice with *Sar1b* allelic disruption showed not only a failure of CM secretion, but also decreased plasma levels of TG, CHOL and HDL-CHOL, all abnormalities observed in CRD patients (22). In the present work, we completed the lipid profiling to include the composition of the different classes of FA in order to examine whether heterozygous mice exhibit fatty acid abnormalities, especially essential FA deficiency. It quickly became apparent that the *Sar1b*^mut/+^ and *Sar1b*^del/+^ mice on HFD presented with more saturated FA, less polyunsaturated FA, a low abundance of n-6, and a reduced SFA/PUFA ratio. More specifically, the *Sar1b*^mut/+^ mice displayed a rise in 16:1(n-7)/18:2(n-6) ratio, an essential FA index deficiency and a decrease in the ratio omega-6/omega-3 (23, 24). Evidence of essential FA deficiency and a reduction of the ratio omega-6/omega-3 were also documented in Canadian and French CRD patients (25).

In congenital malabsorption disorders, not only the quantity of lipoproteins is altered, but also their quality. Our previous studies on hypobetalipoproteinemia and CRD reported irregular lipoprotein composition in patients as reflected by abnormal profile of VLDL, LDL, and HDL (7, 26). In the present work, even with a single disturbed allele, *Sar1b*^mut/+^ and *Sar1b*^del/+^ mice on long-term HFD presented altered lipoprotein composition. However, this alteration is unlikely to be sufficient to explain the significant reduction in HDL-CHOL and non-HDL-CHOL observed in transgenic mice. Indeed, these results are probably underpinned by a reduction in particle number and not just lipoprotein size depending on lipoprotein composition, which may be supported by the slight (although nonsignificant) diminution of HDL ApoA-1. At this time, it is not possible to know whether the changes in lipoprotein composition and size are due to abnormalities in their biogenesis or in their bloodstream metabolism.

Heterozygous *Sar1b* mice were phenotypically normal and have been intercrossed to define the genotype of their progeny. Among the multitude of newborns from heterozygous mating, no homozygous for *Sar1b*^mut/mut^ and *Sar1b*^del/del^ were detected which is not consistent with Mendelian frequency ratios. Thus, we hypothesized that these discrepancies might be due to embryonic lethality as was the case for homozygous murine models with *ApoB* and *MTTP* deletions (27, 28). Monitoring of the cages revealed on rare occasions dead homozygous *Sar1b*^del/del^ pups following *Sar1b*^del/+^ intercrossing, which was not the case for *Sar1b*^mut/+^ mating. Due to the lack of success in finding live births of homozygotes, we subsequently proceeded to identify them during pregnancy. To this end, genomic DNA was isolated from embryos harvested at different stages of embryonic development from *Sar1b*^del/+^ and *Sar1b*^mut/+^ pregnant mice and PCR-genotyped. It was at this point that homozygous embryos (*Sar1b*^del/del^ and *Sar1b*^mut/mut^) were identified at embryonic days 9.5, 13.5, and 18.5. Unlike homozygous embryos in abetalipoproteinemia and hypobetalipoproteinemia (with *ApoB* and *MTTP* deletions, respectively), which developed malformations leading to embryo resorption (27, 28), homozygous *Sar1b*^del/del^ and *Sar1b*^mut/mut^ embryos revealed no macroscopic abnormalities at E18.5. Contrary to homozygous embryos for *ApoB* and *MTTP* deletions, which are thought to be generated by an impediment of the yolk sac to transfer lipids to the embryo, homozygous *Sar1b*^del/del^ and *Sar1b*^mut/mut^ embryos displayed normal embryonic growth and no irregular amounts of lipids were observed in the yolk sacs. Histological examination of the brain and other organs from the homozygous *Sar1b*^del/del^ and *Sar1b*^mut/mut^ embryos did not disclose abnormal phenotypes even if *i*) *Sar1b GTPase* gene expression was abundantly found in the brain (29); *ii*) *Sar1b GTPase* deletion in zebrafish embryos resulted in the absence of neuro-D-positive neurons (30); and *iii*) *Sar1b GTPase* deletion in the developing cerebral cortex impaired radial migration and axon elongation of cortical neurons in mice (31). For the time being, we cannot exclude the possibility that maternal defects in the transfer of fat-soluble vitamins is a potential cause for late death of homozygous embryos with *Sar1b* deletion or mutation. It should also be kept in mind that additional molecular or biochemical abnormalities are most likely behind lethality in the presence of *Sar1b* defects. Sar1b is a ubiquitous protein and it may play crucial roles at multiple sites (29). As indicated by Turgeon *et al.* (32), it is very difficult to determine the cause of prenatal and neonatal death in mice because the complex biological network of relationships between organ defects and physiological processes, which are necessary for mouse survival. For example, skeletal and neuromuscular defects can prevent the embryo from breathing (32). Furthermore, metabolic defects could affect the homeostasis of the fetus and prevent it from surviving as documented by various groups (33, 34, 35).

Although most of the characteristics analyzed in mutant embryos were similar to those of controls, we observed an accumulation of lipid vesicles in the gut of *Sar1b*^del/del^ and *Sar1b*^mut/mut^ homozygous embryos in the absence of fat ingestion. Studies have rarely shown fat deposits in the intestine of mouse embryos following genetic disorders or metabolic perturbations. Nevertheless, maternal diet was shown to trigger lipid accumulation in the liver of embryos. Indeed, exposure of embryos to a HFD increased hepatic TG accretion, oxidative stress, and inflammation (36, 37). In the intestine, a defect of lysosomal lipolysis has been reported to raise prenatal accumulation of lipids, particularly CHOL (38). It is worth noting, however, that maternal lipids were absorbed by the embryonic gut, and even an essential FA-rich diet modified the fatty acid composition of phospholipases in embryonic intestinal plasma membrane (39). Since maternal lipids can be absorbed by the embryo's gut, and as the fetal intestine is endowed with a lipoprotein transport system during embryonic development (40, 41), a defect in intestinal lipid secretion most likely increased lipid accumulation in this organ.

Despite the growing interest in the influence of sex and gender on health, little attention has been paid to congenital malabsorption diseases. Yet, they may influence prevalence, onset, response to treatment and prognosis. In the present study, our results showed that female mice were less affected by HFD and *Sar1b* alterations than male mice. Recently, and in line with our findings, a number of investigations have demonstrated that female rodents were more protected than males against the development of obesity and hepatic steatosis when fed an HFD (42, 43, 44, 45). It is suggested that female mice fed a HFD have a greater ability to use fat as an energy source since they are able to maintain their activity levels and increase their respiratory quotient. This finding is supported by a difference between their catabolic and anabolic neurological signaling pathways (46). It has also been proposed that female mice fed an HFD have a higher energy expenditure than males along with an absence of hyperphagia, leading to protection against the development of obesity (47). With regard to hepatic steatosis, it has been suggested that females accumulate less TG in their livers than males, most likely due to the protective role of estrogen. Female mice in fact produce much more estrogen than males, and estrogen binding to the estrogen receptor-α reduces hepatic lipogenesis and increases FA oxidation (48). This is also supported by the fact that deletion of this receptor in mouse liver increases hepatic lipogenesis (49).

Postprandial lipid clearance in females is also faster and their postprandial peak is lower than in males, most likely due to a higher activity of their lipoprotein lipase (50). These sex differences should not be overlooked in this era of personalized medicine, especially since several studies have reported sex differences in lipid and lipoprotein metabolism (48, 51, 52). Clearly, other useful approaches should be undertaken to understand the mechanisms associated with gender and how the discrepancies affect outcomes and treatments of CRD.

The genotype-phenotype relationship is also an important factor to take into account when engineering genetically modified mice. Noteworthy, the possible presence of allelic heterogeneity indicates that different mutations of the same genetic locus can lead to divergent clinical phenotypes (53). Furthermore, genetic compensation is also a widespread phenomenon that must be taken into consideration (54). The loss of function of a protein triggered by a KO could lead to the upregulation of other genes or proteins that can compensate for this impairment, which explains why some knockdown resulted in more severe phenotypes than those of KO genetic manipulations (54). It was also noted in many diseases that loss-of-function mutations rarely occur in contrast with subtle mutations that are much more common and influence protein properties (55, 56). Therefore, defects in a gene may result in more severe phenotypes or, on the contrary, cause a compensatory mechanism that is not relevant to the disease observed in humans (57). In a previous study, there was genetic compensation with the complete deletion of *Sar1b* in epithelial Caco-2/15 cells (19). This deletion resulted in upregulation of *Sar1a* gene and protein expression, still allowing the secretion of CM despite the genetic defect of *Sar1b GTPase*. Only the double KO of *Sar1a* and *Sar1b* led to the abrogation of lipid secretion. For these reasons, we decided to develop mice with a large base pair deletion and mice with a subtle mutation that was shown to have a severe clinical impact in patients (58). Interestingly, the point mutation of the *Sar1b* gene (described in CRD patients) was more deleterious than the deletion in our mouse models. The exact reasons for these differences are unknown, but allelic heterogeneity or genetic compensation could represent important and influential factors that possibly contributed to our findings since the deletion resulted in the absence of Sar1b GTPase protein production whereas the point mutation led to the formation of an unstable and/or nonfunctional protein (55, 56).

Our last published article reported that mice with altered *Sar1b* can recapitulate some aspects of CRD (12). However, many of these biochemical characteristics were only slightly altered, most likely because heterozygous mice with *Sar1b* defects were only fed a Chow diet with limited fat intake. In the present study, not only did we confirm several previous biochemical factors, but our findings also point out that these genetically modified mice on a HFD are more representative of CRD because they exhibited a lower final body weight, less adipose tissue, and abnormal CHOL concentrations and metabolism in response to intestinal fat absorption.

To determine whether *Sar1b* mutations is associated with a burden on ER protein quality control mechanisms, we evaluated ER stress in the gut and liver. Our findings show that defects of *Sar1b* altered the gene expression of ER stress biomarkers, especially in the intestine of mice on a HFD. In fact, the mRNA levels of key factors such as *PERK*, *IRE1*, *ATF6*, and *GPR78* were increased compared to their respective control. There was possibly an activation of the unfolded protein response in order to recover protein homeostasis via the activation of the PERK, IRE1, and ATF6 sensors, necessary to stimulate downstream pathways (i.e., GPR78) needed to lower protein synthesis while raising ER-associated folding and degradation (59, 60, 61). In sum, our results suggest that *Sar1b* gene defects may generate ER stress and unfolded protein response , indicating that Sar1b is not only a crucial protein for TG-rich lipoprotein secretion. It would have been worthwhile to analyze the protein expression of ER stress factors in addition to their mRNA testing. However, the present work was challenging and quite complex given that we had to evaluate four ER stress factors as a function of two diets (Chow and HFD), two genotypes (*Sar1b*^del/+^ & *Sar1b*^mut/+^), two sexes (male & female) and two organs (intestine and liver). The availability of mouse tissue (particularly intestine) was excessively restraining, making it impossible to support both gene expression and protein mass assays. Despite this, we made a few attempts to explore protein expression, and the variability of results due to the limited number of animals did not allow us to draw any clear conclusions.

In conclusion, this manuscript has shed light on various aspects of the CRD and the Sar1b GTPase protein, which were unknown until now. Our results demonstrate that homozygous alteration of *Sar1b* in mice affects their postnatal viability, while documenting abnormalities in diverse biochemical parameters in association with diet, sex, and *Sar1b* genotype.

## Data availability

All data supporting the findings of this study are contained in the manuscript and in the supplementary file. Raw data can be shared upon request by contacting Nickolas Auclair with the email: auclair.nickolas@gmail.com.

## Supplemental data

This article contains supplemental data.

## Conflict of interest

The funders had no role in study design, decision to publish, or preparation of the manuscript. Therefore, the authors have declared that no competing interests exist.
