# Supplementary material for: High-fat diet reveals the impact of Sar1b defects on lipid and lipoprotein profile and cholesterol metabolism
Source: J Lipid Res. 2023 Aug 7;64(9):100423. doi: 10.1016/j.jlr.2023.100423 (PMC10518719; doi:10.1016/j.jlr.2023.100423)
Supplement: Supplemental data [file mmc1.docx]

**High-fat diet reveals the impact of Sar1b defects on lipid and lipoprotein profile, and cholesterol metabolism**

**Nickolas Auclair^1,2^, Alain T. Sané^1^, Léna Ahmarani^1,3^, Nour-El-Houda Ould-Chikh^1^, Jean- François Beaulieu^4^, Edgard Delvin^1^, Schohraya Spahis^1,3^, Emile Levy^1,2,3*^**

^1^Research Center, CHU Ste-Justine, Departments of ^2^Pharmacology & physiology and ^3^Nutrition, Université de Montréal, Montreal, Quebec, H3T 1C5, Canada**,** ^3^Laboratory of Intestinal Physiopathology, Department of Immunology and Cell Biology, Faculty of Medicine and Health Sciences, Université de Sherbrooke, Sherbrooke, QC J1H 5N4

**SUPPLEMENTARY DATA**

**SUPPLEMENTARY MATERIAL AND METHODS**

**Immunohistochemistry**

Yolk sac cryosections from WT, Sar1b^del/del^, and Sar1b^del/+^ mice, as well as Sar1b^mut/+^ and Sar1b^mut/mut^ mice were cut at 4-6 µm and stored at -20°C. On the day of immunofluorescent staining, the slides were transferred to room temperature for 30 minutes, fixed with 4% PFA for 10 minutes and washed with PBS. They were then incubated with blocking solution (1% BSA, 3% NGS, 1% Triton X-100 in PBS) for 60-90 minutes at room temperature. The slides were then incubated for 2 hours with primary antibodies diluted in 1% BSA, 1% NGS, 0.1% Triton X-100 in PBS). After this incubation, the slides were washed three times with PBS and transferred with goat anti-rabbit secondary antibody for 1 to 1.5 hours. The slides were finally washed three times with PBS, dried and mounted with Gelmount (Sigma-Aldrich, USA). The list of antibodies and their dilution used for these experiments can be found **in Table S2**.

**Table S1: List of all primers used for RT-qPCR analysis**

|  | **Primers** |
| --- | --- |
| ***Abcg8*** | Forward: 5-GCACTGGTCATGGCTGAGAA-3’ |
|  | Reverse: 5-CACAGGAGTCTTGGCTGCTA-3 |
| ***Mttp*** | Forward: 5-ATGATCCTCTTGGCAGTGCTT-3 |
|  | Reverse: 5-TGAGAGGCCAGTTGTGTGAC-3 |
| ***Ldlr*** | Forward: 5-GCTCCATAGGCTATCTGCTCTTCA-3 |
|  | Reverse: 5-CTGCGGTCCAGGGTCATC-3 |
| ***Abca1*** | Forward: 5-AGGGTTTCTTTGCTCAGATTGTC-3 |
|  | Reverse: 5-TGCCAAAGGGTGGCACA-3 |
| ***Sr-b1*** | Forward: 5-TCAGAAGCTGTTCTTGGTCTGAAC-3 |
|  | Reverse: 5-GTTCATGGGGATCCCAGAGA-3 |
| ***Npc1l1*** | Forward: 5-CCACAGACCCTGTGGAACTG-3  Reverse: 5-GCTCGTCATGGAAAGCCTTT-3 |
| ***Actin*** | Forward: GACAGGATGCAGAAGGAGATTACTG  Reverse: CCACCGATCCACACAGAGTACTT |
| ***Hmg-Coar*** | Forward: 5-ATTCTGGCAGTCAGTGGGAACT-3  Reverse: 5-CCTCGTCCTTCGATCCAATTTA-3 |
| ***Pcsk9*** | Forward: 5-GCACCAGACAGAGGAAGACC-3  Reverse: 5-GTGACCCTGCCCTCAATCT-3 |
| ***Srebp2*** | Forward: 5-CGACCAGCTTTCAAGTCCTG-3  Reverse: 5-CCTGTACCGTCTGCACCTG-3 |
| ***Lxrα*** | Forward: 5-GGAGTGTCGACTTCGCAAAT-3  Reverse: 5-CTTGCCGCTTCAGTTTCTTC-3 |
| ***Perk*** | Forward: 5-ATCTGTTCTGCCTTGGGATG-3  Reverse: 5-CAAAGTGGCCAACACTGAAA-3 |
| ***Gpr78*** | Forward: 5-TGCAGCAGGACATCAAGTTC-3  Reverse: 5-TACGCCTCAGCAGTCTCCTT-3 |
| ***Ire1*** | Forward: 5-CGCATCACCAAGTGGAAGTA-3  Reverse: 5-CCTTCCAGCAAAGGAAGAGT-3 |
| ***Atf6*** | Forward: 5-TGTCACTGGTCCTGGAAACA-3  Reverse: 5-TGAATGATGATGGCTTTTGC-3 |

**Table S2: List of all antibodies used for immunohistochemistry analysis**

| **Antibody** | **Company** | **Type** | **Dilution** |
| --- | --- | --- | --- |
| Anti-ApoB | Proteintech #20578-1-AP | Rabbit anti human | 1:20 |
| Anti-MTTP | Dr Jean Davignon | Rabbit anti mouse | 1:20 |
| Anti-Rabbit IgG – Alexa Fluor 488 | Invitrogen # A-11008 | Goat anti rabbit | 1:500 |

**SUPPLEMENTARY RESULTS**


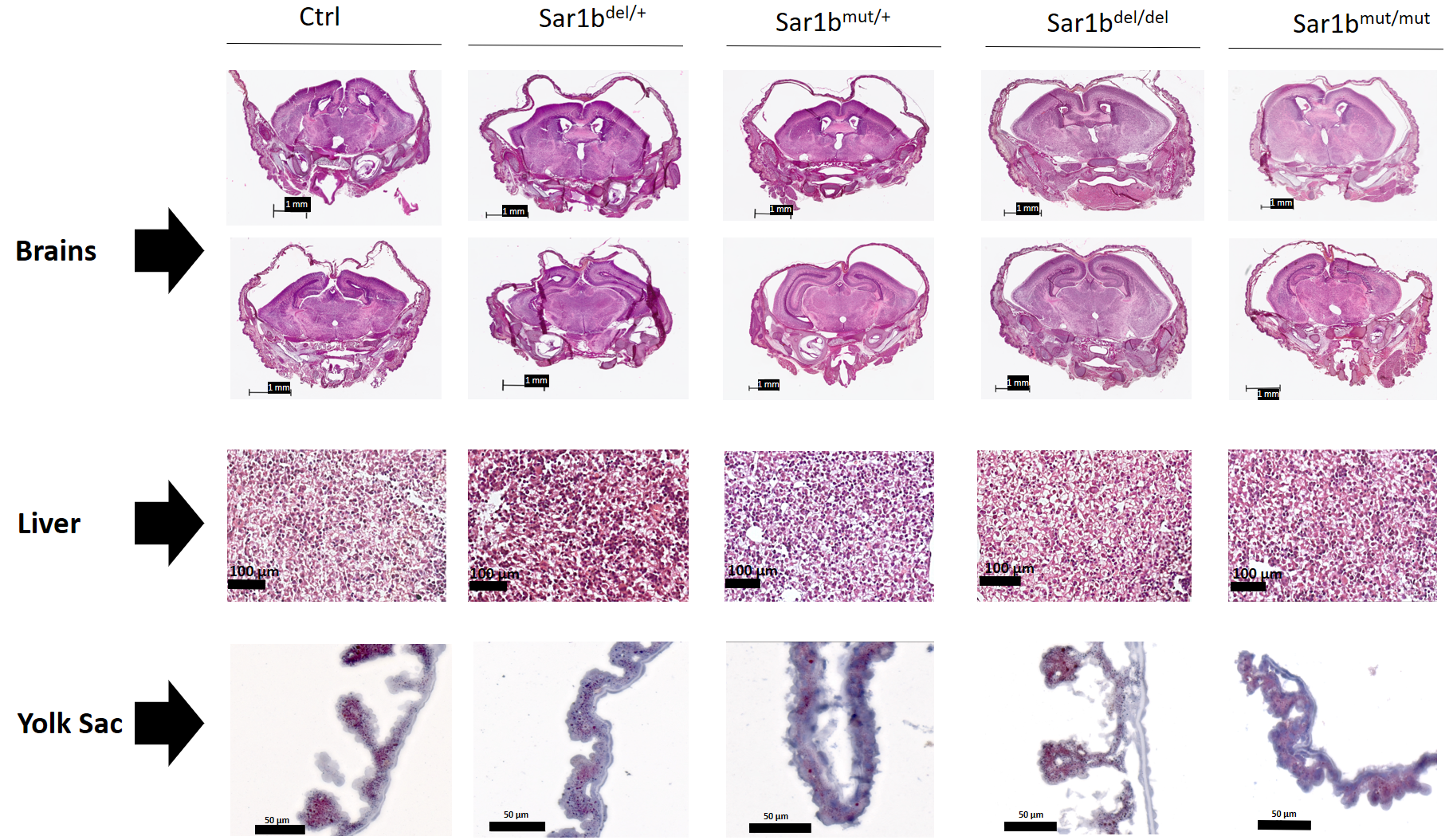


**Figure S1** **Histology of brain, liver and yolk sac of Sar1b^del/+^ and Sar1b^mut/+^ mouse embryos.** Heterozygous male and female Sar1b^del/+^ and Sar1b^mut/+^ mice were bred. As soon as a vaginal plug was found, the female mice were considered pregnant at day E0.5. After 18 days, the female mice were euthanized with a CO_2_ stream and the embryos were collected. Livers and brains were isolated from the embryos at E18.5, fixed with formalin and embedded in paraffin. After staining with hematoxylin and eosin, photographs of these brains and livers were taken under the microscope. Yolk sacs were taken from mice at 13.5 days of age, fixed in formalin and embedded in O.C.T. Photographs of these membranes were also taken under the microscope and stained with Oil Red O and hematoxylin. At least two specimens per group were used for specified analyses.


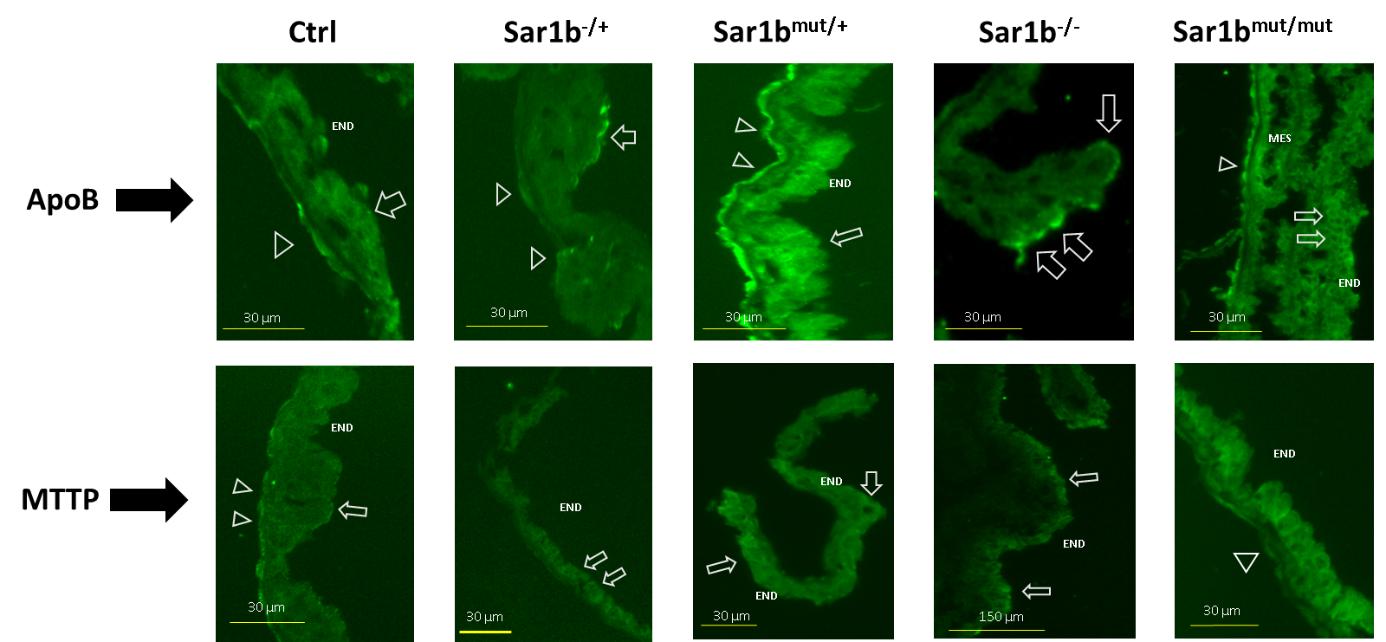


**Figure S2 Immunostaining of ApoB and MTTP in the yolk sac of Sar1b-disrupted mice.**

Male and female heterozygous Sar1b^del/+^ and Sar1b^mut/+^ mice were mated. Once a vaginal plug was found, female mice were considered pregnant at day E0.5. Yolk sacs were collected from mice at 13.5 days of age, fixed in formalin and embedded in O.C.T. Cryosections of the yolk sacs were cut at 4-6 µM for ApoB and MTTP immunostaining as described in Materials and Methods. This figure contains examples of images obtained after labeling, with the location of endodermal cells (END) and arrows indicating the presence of labeling. At least two specimens per group were used for the specified analyses.
